# Editorial: Cell death: from its induction to the removal of dying cells!

**DOI:** 10.3389/fcell.2023.1195775

**Published:** 2023-04-11

**Authors:** Maria C. Tanzer, Bart Tummers, Ivan K. H. Poon

**Affiliations:** ^1^ Department of Medical Biology, Walter and Eliza Hall Institute of Medical Research, The University of Melbourne, Parkville, VIC, Australia; ^2^ Centre for Inflammation Biology & Cancer Immunology, Department of Inflammation Biology, School of Immunology & Microbial Sciences, King’s College London, London, United Kingdom; ^3^ Department of Biochemistry and Chemistry, La Trobe Institute for Molecular Science, Research Centre of Extracellular Vesicles, La Trobe University, Melbourne, VIC, Australia

**Keywords:** efferocytosis, cell death, inflammation, macrophages, cytokines

This Research Topic aims to bring attention to the latest advancements in the study of cell death and the clearance of dead cells. Although extensive research has been conducted on understanding the signalling pathways involved in cell death, less is known about the mechanisms that drive the recruitment of macrophages towards dead cells and the efficient engulfment of cell corpses. As evidenced by numerous clinical trials investigating drugs that target cell death in various diseases, there is comparatively less emphasis on targeting the clearance of dead cells. This Research Topic explores novel aspects of research in cell death and dead cell removal, strategies for controlling it during inflammatory disease and highlights the importance of understanding the complex mechanisms driving dead cell clearance in health and disease.

Pontejo et al. present their recent findings that chemokines important for attracting macrophages to clear dead cells can bind to phosphatidylserine (PS) exposed on dead cells. Their proposed “breadcrumb” model suggests that upon cell death, cellular debris is dispersed in a gradient-like manner around the dead cell. Supporting this concept, they highlighted a previous study that showed that certain chemokines could bind to PS exposed on cellular debris. The chemokine-PS binding is, therefore, a potent mechanism to attract macrophages to the site of the dead cell’s body, thus aiding in its clearance.

Yuan et al. describe the importance of ubiquitin complexes in various aspects of cell death signalling and during the removal of dead cells. They review the key ubiquitin systems regulating these processes and stress the need to further investigate the role of ubiquitination and deubiquitinases in dead cell clearance.

An alternative strategy for managing inflammatory diseases involves suppressing the production of pro-inflammatory cytokines without directly modulating cell death or dead cell clearance. A study conducted by McHugh et al. proposes CDK9 inhibition as a promising strategy for developing novel anti-inflammatory therapy. The inhibition of this transcriptional cyclin-dependent kinase was found to significantly reduce the production of pro-inflammatory cytokines by macrophages following LPS infection. Importantly, this reduction in cytokine production occurred without any significant increase in cell death or impairment of macrophages’ phagocytic function. Conversely, overexpressing CDK9 in THP-1 cells led to increased pro-inflammatory cytokine production compared to wild-type cells. These findings suggest that it may be possible to interfere with transcriptional processes using CDK9 inhibition without impacting macrophages’ essential cellular functions.

Controlling the clearance of dead cells is crucial for maintaining tissue homeostasis and perturbation of this process can lead to pathological conditions. This is especially relevant in autoimmune diseases such as rheumatoid arthritis, diabetes, multiple sclerosis, and systemic lupus erythematosus, where impaired dead cell removal has been implicated. Ge et al. provide a comprehensive overview of the role of various cellular systems in dead cell clearance and its association with infection and inflammatory disorders.

In order to successfully treat inflammatory and infectious diseases by regulating cell death and dead cell removal, it is crucial to have a precise understanding of the underlying mechanisms. Tanzer’s minireview highlights the different steps involved in dead cell removal, from engulfment to digestion, and emphasises the potential areas where these processes can be disrupted. Additionally, the review highlights the potential impact of dead cell cargo on the macrophage programming that occurs during dead cell clearance.

Our Research Topic has unveiled the complex nature of dead cell removal, revealing key knowledge gaps that necessitate further exploration. A comprehensive understanding of cell death and clearance is crucial for gaining precise insights into the underlying molecular mechanisms that drive inflammation, cancer, autoimmune disorders, and infectious diseases. This knowledge is essential for developing successful therapeutic targets and advancing our ability to control these diseases.

